# *Chlamydia trachomatis* genovar distribution in clinical urogenital specimens from Tunisian patients: high prevalence of *C. trachomatis* genovar E and mixed infections

**DOI:** 10.1186/1471-2334-12-333

**Published:** 2012-11-30

**Authors:** Houda Gharsallah, Olfa Frikha-Gargouri, Hanen Sellami, Fatma Besbes, Abir Znazen, Adnene Hammami

**Affiliations:** 1 Department of Microbiology and research laboratory “Microorganismes et Pathologie Humaine”, Habib Bourguiba university hospital, medical school of Sfax, University of Sfax, Sfax, Tunisia; 2 Biopesticides Team, LPAP, Sfax Biotechnology Centre, University of Sfax, Sfax, Tunisia; 3 Mailing address: Laboratory of microbiology, University school of medecine of Sfax, Avenue Magida Boulila, 3027 Sfax, Tunisia

**Keywords:** Chlamydia trachomatis, Genotyping, Reverse hybridization method

## Abstract

**Background:**

This epidemiological study was carried out in Sfax (south of Tunisia) and focused on genital *Chlamydia trachomatis* (*C. trachomatis*) genovar distribution.

**Methods:**

One hundred and thirty seven genital samples from 4067 patients (4.2%) attending the Habib Bourguiba University hospital of Sfax over 12 years (from 2000 to 2011) were found to be *C. trachomatis* PCR positive by the Cobas Amplicor system. These samples were genotyped by an in house reverse hybridization method.

**Results:**

One hundred and eight (78.8%) samples contained only one genovar and 29 (21.2%) samples contained two or three genovars. Genovar E was the most prevalent (70.8%) single genovar and it was detected in 90.6% of all the cases. Genovars J, C and L1-L3 were not detected in our samples whereas ocular genovars A and B were in 5 cases. All the five cases were mixed infections. Men had more mixed infections than women (*p*=0.02) and were more frequently infected by genovars F and K (*p*<0.05). No associations between current infection, infertility and the genovar distribution were observed. Patients coinfected with *Neisseria gonorrhoeae* were also significantly more frequently infected with mixed genovars (*p*=0.04).

**Conclusions:**

In conclusion, we have reported a high prevalence of genovar E and of mixed infections in our study population. Such data could have implications for the control and vaccine development of *C. trachomatis* in Tunisia.

## Background

*Chlamydia trachomatis* (*C. trachomatis*) infections are the most common sexually transmitted bacterial infections in the world. The World Health Organization estimated that 92 million new cases of *Chlamydia* infections occurred globally in 2001 [1]. Genital infection with *C. trachomatis* is associated with a wide spectrum of diseases if the infection was not treated. In men, urogenital *C. trachomatis* infection causes urethritis that may lead to complications such as epididymitis in rare cases. In women, clinical manifestations show cervicitis that can ascend in the upper genital tract and cause pelvic inflammatory disease and infertility. The diseases caused by *C. trachomatis* also include trachoma, the world’s leading cause of preventable blindness [2] as well as lymphogranuloma venereum (LGV), a severe systemic infection [3].

*C. trachomatis* strains are conventionally divided into 19 genovars based on the heterogeneity of the *ompA* gene sequence. The different genovars of *C. trachomatis* are associated with the various clinical manifestations caused by this bacterium. Genovars A to C are predominantly associated with trachoma and were rarely detected in urogenital diseases. Genovars D to K are associated with urogenital diseases, and genovars L1 to L3 are associated with the LGV. *C. trachomatis* genovars are also classified into 3 distinct groups on the basis of their amino acid sequence homology: the B group (B, Ba, D, Da, E, L1, L2a and L2b), the C group (A, C, H, I, Ia, J, Ja, K and L3) and the intermediate group (F and G) [4].

In the previous studies performed in Tunisia, *C. trachomatis* was reported in 15% among men with urethritis (data not published), in 59% among patients with arthritis [5], in 43.3% among infertile men with leukocytospermia [6] and in 73% of prostitutes [7]. Thus, *C. trachomatis* infection seems to be prevalent in our country, but no information about *C. trachomatis* genovar distribution in our population has been described so far.

Thus, we undertook this study to genotype *C. trachomatis* strains circulating in Tunisia using our in house reverse hybridization method. We also aimed to assess the relationship between gender, age, clinical and microbiological factors and *C. trachomatis* genovars. Such data might give insights into the *C. trachomatis* strains circulating in Tunisia and help develop strategies of sexually transmitted infections control.

## Methods

### Clinical *C. trachomatis* samples

One hundred and seventy two urethral and/or endocervical swabs positive for *C. trachomatis* using Cobas Amplicor (Roche molecular system, Mannheim Germany), were collected from Tunisian patients at the Habib Bourguiba University hospital in Sfax between February 2000 and June 2011. Specimens were collected using cytobrush, and the contents were transferred immediately into a tube containing 1ml 2-SP transport medium. Specimens were kept at −80°C until testing.

These specimens were also tested for *Neisseria gonorrhoeae* (*N. gonorrhoeae*) nucleic acids with the Amplicor CT/NG polymerase chain reaction (PCR). DNA extraction from 200μl 2-SP transport medium and PCR procedures were performed according to the manufacturer’s instructions.

All subjects provided verbal informed consent, and the study protocol was approved by our ethics committee (Association d’Enregistrement et de Lutte Contre le Cancer du Sud Tunisien).

### Extraction of genomic DNA

Genomic DNA from clinical swabs was extracted from 200 μl of urethral and endocervical swabs using the QIAmp DNA Mini kit (QIAGEN GmbH, Hilden, Germany) according to the manufacturer’s instructions. The DNA was then eluted in 50 μl of the elution buffer and stored at −20°C. The extracted DNA was tested for human ß-globin gene to check for PCR inhibitors in the samples. Primers ß-GPCO (5′-ACACAACTGTGTTCACTAGC- 3′), and ß-GPCPO (5′-GAAACCCAAGAGTCTTCTCT- 3′), were used to amplify a 209 bp fragment of the human ß-globin gene.

### PCR amplification of the VS1-VS2 fragment

Urethral and endocervical DNA positive by Cobas Amplicor *C. trachomatis* test were selected for the *ompA* amplification and genotyping. A semi nested PCR was used for the amplification using the NLO (Biotin- ATGAAAAAACTCTTGAAATCG) [8] and C214 (Biotin-TCTTCGAYTTTAGGTTTAGATTGA) [9] primers for the first PCR and the NLO and CT4 (Biotin-GATTGAGCGTATTGGAAAGAAGC) [10] primers for the semi nested PCR. The reaction mixture consisted of 3μl of extracted DNA, 0.5μM of each primer, 0.2 mM dNTP; 1U of the flexi Taq DNA polymerase (Promega, Madison WI, USA). PCRs were performed using the GeneAmp PCR System 9600 thermocycler under the following conditions: 2 min of denaturation at 95°C, followed by 35 cycles of amplification at 95°C for 45 sec, 45°C for 45 sec, and 72°C for 45 sec. The *ompA* PCR products were visualized after electrophoresis in 1% agarose gels by ethidium bromide staining.

### The reverse hybridization method

An in house reverse hybridization method for *C*. *trachomatis* genotyping was first developed in our laboratory and was validated using 14 *C*. *trachomatis* reference strains [11]. Then this method was used for the specific detection of *C*. *trachomatis* genotypes. Briefly, the nylon membrane was spotted with probes hybridizing specifically to genovars A to L3 at specific positions. Reverse hybridization probes were then attached to the membrane by baking at 120°C for 30 min. Membranes were prehybridized for 30 min at 37°C and then hybridization took place over the night at 37°C with shaking. The biotinylated duplex DNA-probe was revealed after incubation with the alkaline phosphatase enzyme for one hour at 37°C and then with its substrate: the Western Blue stabilized substrate (Promega, Madison WI, USA) for 30 min at room temperature and in the dark. Washing was performed between each of the incubation steps.

### Statistical analysis

Data were analyzed using SPSS version 13.0. Differences were statistically compared by the Chi-square test or the Fisher’s exact test when sample sizes were small. *p* < 0.05 was considered as significant.

## Results

During twelve years, urethral and endocervical swabs from 4067 Tunisian patients were collected in the Habib Bourguiba University hospital in Sfax between February 2000 and June 2011 and were subjected to the Cobas Amplicor CT/NG PCR testing. One hundred and seventy two samples (4.2%) were found to be PCR positive and only 138 were available for *C. trachomatis* genotyping. Of these 138 samples, 84 (60.9%) were collected from men and 54 (39.1%) from women. Data on age were obtained from 115 individuals. The median age was 30 years and the 95% confidence interval was [28.85-31.45]. Thirty two individuals (23.9%) were under 25 years. Thirty seven individuals (27%) were consulted for infertility (5 men and 32 women), 96 (70%) for the current infection (76 men and 20 women) and one man consulted for both infertility and the current infection (0.7%). Data were not available for the remaining 3 patients (2.2%). Mixed infections with *N. gonorrhoeae* were detected in 18 cases (13.1%) (Table 1).

**Table 1:** Genovar types according to patient characteristics

**Genovar**	**Total** **N = 137 (%)**	**Total** **N = 137 (%)**		***p *value**	Current infection * N = 96 (%)		***p *value**	Infertility * N = 37 (%)		***p *value**	**NG coinfection** **N = 18 (%)**		***p *value**
**Gender**		**Men** **N = 84 (%)**	**Women** **N = 53 (%)**		**Men** **76 (57.1)**	**Women** **20 (15)**		**Men** **5 (3.6)**	**Women** **32 (24)**		**Men** **16 (11.7)**	**Women** **2 (1.5)**	
**Single**	108 (78.8)	61 (72.6)	47 (88.7)	**0.03**	54 (40.6 )	18 (13.5)	0.14	5 (3.6)	28 (21)	1.00	9 (8.0)	2 (1.5)	0.49
**D**	2 (1.5)	0 (0)	2 (3.8)	0.14	0 (0)	2 (1.5)	**0.04**	0 (0)	0 (0)	Nd	0 (0)	0 (0)	Nd
**E**	97 (70.8)	55 (65.5)	42 (79.2)	0.12	49 (36.8)	14 (10.5)	0.79	4 (3)	27 (20.3)	1.00	7 (6.5)	2 (1.5)	0.47
**F**	4 (2.9)	4 (4.8)	0 (0)	0.16	3 (2.2)	0 (0)	1.00	1 (0.7)	0 (0)	0.14	1 (0.7)	0	1.00
**G**	3 (2.2)	0 (0)	3 (5.7)	0.06	0 (0)	2 (1.5)	**0.04**	0 (0)	1 (0.7)	1.00	0 (0)	0 (0)	Nd
**H**	1 (0.7)	1 (1.2)	0 (0)	1.00	1 (0.7)	0 (0)	1.00	0 (0)	0 (0)	Nd	0 (0)	0 (0)	Nd
**K**	1 (0.7)	1 (1.2)	0 (0)	1.00	1 (0.7)	0 (0)	1.00	0 (0)	0 (0)	Nd	1 (0.7)	0 (0)	1.00
**Multiple**	29 (21.2)	23 (27.4)	6 (11.3)	**0.03**	22 (16.1)	2 (1.5)	0.14	0 (0)	4 (3)	1.00	7 (5.1)	0 (0)	0.49
**A + E**	1 (0.7)	1 (1.2)	0 ( 0)	1.00	1 (0.7)	0 (0)	1.00	0 (0)	0 (0)	Nd	0 (0)	0 (0)	Nd
**B + E**	2 (1.5)	0 (0)	2 (3.8)	0.15	0 (0)	1 (0.7)	0.21	0 (0)	1 (0.7)	1.00	0 (0)	0 (0)	Nd
**D + F**	1 (0.7)	1 (1.2)	0 (0)	1.00	1 (0.7)	0 (0)	1.00	0 (0)	0 (0)	Nd	0 (0)	0 (0)	Nd
**E + F**	5 (3.6)	4 (4.8)	1 (1.9)	0.65	4 (3)	0 (0)	0.58	0 (0)	1 (0.7)	1.00	0 (0)	0 (0)	Nd
**E + G**	4 (2.9)	4 (4.8)	0 (0)	0.16	4 (3)	0 (0)	0.58	0 (0)	0 (0)	Nd	2 (1.5)	0 (0)	1.00
**E + H**	5 (3.6)	4 (4.8)	1 (1.9)	0.65	4 (3)	0 (0)	0.58	0 (0)	1 (0.7)	1.00	2 (1.5)	0 (0)	1.00
**E + K**	3 (2.2)	3 (3.6)	0 (0)	0.28	3 (2.2)	0 (0)	1.00	0 (0)	0 (0)	Nd	0 (0)	0 (0)	Nd
**E + I**	1 (0.7)	1 (1.2)	0 (0)	1.00	0 (0)	0 (0)	Nd	0 (0)	0 (0)	Nd	1 (0.7)	0 (0)	1.00
**A + E + F**	1 (0.7)	0 (0)	1 (1.9)	0.39	0 (0)	0 (0)	Nd	0 (0)	1 (0.7)	1.00	0 (0)	0 (0)	Nd
**B + D + H**	1 (0.7)	1 (1.2)	0 ( 0)	1.00	1 (0.7)	0 (0)	1.00	0 (0)	0 (0)	Nd	1 (0.7)	0 (0)	1.00
**E + F + H**	1 (0.7)	1 (1.2)	0 ( 0)	1.00	1 (0.7)	0 (0)	1.00	0 (0)	0 (0)	Nd	0 (0)	0 (0)	Nd
**E + H + K**	4 (2.9)	3 (3.6)	1 (1.9)	1.00	3 (2.2)	1 (0.7)	1.00	0 (0)	0 (0)	Nd	1 (0.7)	0 (0)	1.00

Statistically significance is marked in bold.

* The status of infection data was not available for 3 patients. The remaining patient, presenting both current infection and infertility, was discarded from the analysis.

Nd: Not determined.

All the 138 samples *C. trachomatis* Cobas PCR positive were successfully screened by the *β*-globin PCR test and no inhibition was detected. One hundred and thirty seven samples were then amplified by our semi nested PCR, generating a sensitivity of 99%. These amplicons were used for the detection of *C. trachomatis* genotypes by the reverse hybridization method. Genovar distribution results of the 137 samples are shown in Table 1. A heterogeneous distribution of the 137 *C. trachomatis* genovars was observed. All genovars belonging to the D–K urogenital group were detected except the genovar J. One hundred and eight samples (78.8%) were infected with a single genovar. The remaining 29 samples (21.2%) showed mixed infections. Genovar E was the most prevalent (70.8%) single infection. In single and mixed infections together, genovar E was detected in 90.6% of cases (124/137). The most prevalent mixed infection was observed to be genovars E and F, and E and H. Genovar B was detected in mixed infection with genovar E in two cases and with genovars D and H in one case. Genovar A was detected in mixed infections with genovar E in one case and with genovars E and F in one case. Genovar C and LGV genovars were not detected in our urogenital samples. The genovar distribution was also investigated according to the year of collection of samples (Additional file 1: Table S1). Generally, all single and mixed genotypes were distributed all over the 12 years.

The relationships between gender, age, current *C. trachomatis* infection, infertility, *N. gonorrhoeae* infection data and *C. trachomatis* genotypes or group results were examined (Table 2). No statistical differences were observed for the distribution of *C. trachomatis* genotypes between men and women (*p*<0.05). However, significantly more men had mixed infections (*p*=0.02) than women. Genovars F and K were observed more frequently in men than in women (*p*<0.05) too. In fact, 10.7% and 3.7% of men and women, respectively, were infected with genovar F and 8.3% and 1.8% respectively with genovar K. No association was observed between age, current infection, infertility and the genovar distribution. Patients infected with *N. gonorrhoeae* were also significantly more frequently infected by mixed infection (*p*=0.04), and by genovars other than E alone (*p*=0.03) (Table 2).

**Table 2:** **Factors associated with *C. trachomatis *genotype**

		**Genotype**					
		**Single** **N (%)**	**Mixed** **N (%)**	**E single** **N (%)**	**E associated** **N (%)**	**E single** **N (%)**	**Other genotypes** **N (%)**
**Gender**	Men (84)	61 (56.5)	23 (79.3)	55 (56.7)	21 (77.8)	55 (56.7)	29 (72.5)
	Women (53)	47 (43.5)	6 (20.7)	42 (43.3)	6 (22.2)	42 (43.3)	11 (27.5)
***p***		**0.02**		**0.04**		NS	
Age *	<= 25 (32)	22 (24.4)	10 (40)	20 (24.4)	9 (39.1)	20 (24.4)	12 (36.4)
	>25 (83)	68 (75.6)	15 (60)	62 (75.6)	14 (60.9)	62 (75.6)	21 (63.6)
***p***		NS		NS		NS	
**Infertility ‡**	Presence (37)	33 (24.8)	4 (30)	31 (23.3)	4 (30)	31 (23.3)	6 (4.5)
***p***		NS		NS		NS	
**Current infection ‡**	Presence (96)	72 (54.1)	24 (18)	63 (47.3)	17 (12.8)	63 (47.3)	33 (24.8)
***p***		NS		NS		NS	
**NG coinfection**	Presence (18)	11 (10.2)	7 (24.1)	9 (9.3)	6 (22.2)	9 (9.3)	9 (22.5)
***p***		**0.04**		NS		**0.03**	

Statistically significance is marked in bold.

* The age data were available for only 115 patients.

**‡**The status of infection was not available only for 3 patients. One patient, presenting both current infection and infertility, was discarded from the analysis.

## Discussion

Many studies have reported the epidemiology of *C. trachomatis* infection throughout the world. The prevalence of *C. trachomatis* infections varies from 2% to 5.6% in selected and unselected populations [12]. In line with previous studies, the prevalence of *C. trachomatis* infection in our city of Sfax was found to be 4.2%. To our knowledge, *C. trachomatis* genovar distribution has not been reported before in our country. *C. trachomatis* genotyping was investigated in all urogenital samples collected in Sfax (Tunisia) found to be Cobas amplicor PCR positive since 2000. Ninety nine percent (137/138) of our samples were successfully amplified using our semi nested PCR. This sensitivity is high when compared with those reported in other studies ranging from 44 to 99% [10,13-15].

The reverse hybridization used in our study is simple and fast. It requires low technology to be performed. It is powerful in the diagnosis of mixed infection and did not show any cross reactivity between reference strains.

In our study, genotype E was the most prevalent being detected in 70.2% of single infections. All the other genotypes were detected at percentages ranging from 0.7 to 2.2%. When considering single and mixed infections, genovar E was detected in 92.6% of the cases followed by genovar F that was detected in 10.3% of the cases. The detection of genovar E was remarkably high in our study but was in line with that of Gita *et al.,*[13] performed in New Delhi (India) reporting the detection of only genovar E in 22 specimens positive for *C. trachomatis* by PCR-RFLP. Their high percentage was explained by the small number of specimen processed which was not so in our case. According to the literature, genovars D, E, F and G were reported to be the most prevalent genovars worldwide [8,9,16-21]. Differences in the relative proportions of these genovars in different geographical areas were reported. For example, in Cambodia, genovar D (22.2%) was the most prevalent followed by genovar F (18.5%) [22]. In Australia, the most common genovar was E (41%) followed by genovar F (26%) [23]. In Sweden, the most common genovar was E (47%) followed by genovar F (17%) [15]. Differences in genotype prevalence were found in different study populations. In Thailand, genovar F was the most prevalent genotype (60%) among non sex workers, but decreased to 29% among sex workers [24]. The high detection rate of genovars D, E, F, and G could be related to a longer persistence time of their infections than the other genovars in the genital tract [25].

The high prevalence of genovar E in our study could be explained by a specific immune status of Tunisian patients and by distinctive characteristics of some genotype E strains in our country. Such strains could not be distinguished by our reverse hybridization method. Thus, the application of new typing schemes based on more discriminative method such as multilocus sequence typing or multiple loci variable number of tandem repeats analysis might provide a better understanding of our high prevalence of *C. trachomatis* genovar E. An interesting issue would be to further examine whether all or most of the genovar E in our patients were of the same strain or not. Furthermore, an assessment of *in vitro* studies of infectivity and cytotoxicity of our local strains in urogenital cell lines may also help to explain the high prevalence of genovar E in our population. Jones *et al.,*[26] suggested that genovar E can outcompete other genovars for nutrients and growth factors leading to a rapid expansion of genovar E when compared to the other genovars.

Mixed infections were detected in our study in 21.1% of the cases. This percentage seems to be high as most of mixed infections in the literature occur at a percentage not exceeding 15% [19,27-30]. Mixed infections may result from two separate episodes of infection and the lack of cross protection between genotypes [14]. In our study, single genovars were rarely detected except for genovar E. Genovars H and K were detected once in single infections but were detected at high frequencies in mixed infections (11 and 7 times, respectively). These genovars are probably relatively low grade pathogens which could only persist in the presence of other genovars [31].

Trachoma genovars A and B were also detected in 5 patients (3.6%). All of them were observed in mixed infections. This could not be due to cross reactivity since our technique was well studied with a mixture of reference strains. Also, these genovars were involved in urogenital infections [32-37].

The genovar detection in our study was similar among men and women in line with previous studies suggesting that specific serotypes do not preferentially infect one gender [23,38]. However, the occurrence of mixed infections is significantly higher among men than women. Furthermore, a statistically significant difference was found for the occurrence of genovars F and K which were observed more frequently in men than in women. Men are probably sexually active with more than one partner leading to the occurrence of mixed infections and infection with another genovar than E. No statistically significant difference in distribution of genotypes and age was found such a result is in a total agreement with other studies [39].

In our study, no association was found between *C. trachomatis* genotype E and the patient’s infection status (current symptomatic infection and infertility). According to the literature, genovar E was reported at high frequencies in symptomatic adults [40] as well as in asymptomatic adults [16,18,19]. *C. trachomatis* genovar E was also found in our study to be the most prevalent genovar. In infertile patients from Mexico, *C. trachomatis* genovar F was the most prevalent genovar (54%) followed by genovars E, G, K and LGV (9%) [41].

Patients with *N. gonorrhoeae* infection were significantly more frequently infected by mixed genovars and by genovars other than the single genovar E (Table 2). According to the literature, little is known about *N. gonorrhoeae* coinfection and its association with *C. trachomatis* genovars. Papadogeorgakis *et al.,*[36] reported a significantly higher proportion of *N. gonorrhoeae* coinfections among patients with urethritis with genovariant Ja. No other associations were reported except for the significant association of *N. gonorrhoeae* coinfection with genovar D [42].

Yet, we could mention a few limitations of our study. First, the smaller sample size of infected patients could affect the relationship between *C. trachomatis* genotypes and clinical manifestations. Second, supplementary information about behavioral characteristics would be useful to explain the difference in genotype distribution among men and women. In addition, the reverse hybridization method used in this study could not detect new genovars and recombinant clinical strains. These could lead to cross hybridizations [11]. The presence of trachoma genotypes and the high frequency of mixed infections have to be confirmed by cloning and complete *ompA* gene sequencing.

## Conclusion

In conclusion, the present study is the first description of *C. trachomatis* genovar distribution in Tunisia. *C. trachomatis* genotype E infection rate was found to be considerably much higher than other reports in the world. We have also detected a high frequency of mixed infections. This genotype distribution is unique and further studies focused on specific groups are needed to assess whether this figure is specific to Tunisia only. The data obtained in this study could have implications for the control and vaccine development of *C. trachomatis* in Tunisia.

## Competing interests

The authors declare that they have no competing interests.

## Authors’ contributions

HG carried out all the studies, analyzed the data and drafted the manuscript. OFG helped with the analysis, discussion of the data and correction of the manuscript. FB and HS helped with the analysis and discussion of the data. AZ helped with the discussion of the data. AH participated in the study design and helped to draft the manuscript. All authors have read and approved the final manuscript.

## Pre-publication history

The pre-publication history for this paper can be accessed here:

http://www.biomedcentral.com/1471-2334/12/333/prepub

## Supplementary Material

Additional file 1: Table S1 The prevalence of *C. trachomatis *in Tunisian patients. Click here for file
