# Supplementary material for: Chlamydia trachomatis genovar distribution in clinical urogenital specimens from Tunisian patients: high prevalence of C. trachomatis genovar E and mixed infections
Source: BMC Infect Dis. 2012 Nov 30;12:333. doi: 10.1186/1471-2334-12-333 (PMC3573954; doi:10.1186/1471-2334-12-333)
Supplement: Additional file 1: Table S1 — The prevalence of C. trachomatis in Tunisian patients. [file 1471-2334-12-333-S1.doc]

| **Study year** | **02-2000** | **2001** | **2002** | **2003** | **2004** | **2005** | **2006** | **2007** | **2008** | **2009** | **2010** | **06-2011** | **total** |
| --- | --- | --- | --- | --- | --- | --- | --- | --- | --- | --- | --- | --- | --- |
| **Samples** | **125** | **284** | **410** | **413** | **341** | **433** | **321** | **451** | **410** | **468** | **332** | **79** | **4067** |
| **Cobas positive (%)** | 8  (6.4) | 10  (3.5) | 18  (4.4) | 18  (4.4) | 7  (2.1) | 11  (2.5) | 13  (4.0) | 35 *****  (7.8) | 18  (4.4) | 19  (4.1) | 13  (3.9) | 2  (2.5) | 172  (4.2) |
| **genotypes positive (%)** | 2  (25.0) | 3  (30.0) | 15  (83.3) | 10  (55.6) | 5  (71.4) | 11  (100) | 12  (92.3) | 31  (88.6) | 16  (88.9) | 17  (89.5) | 13  (100) | 2  (100) | 137  (79.7) |
| **D** |  |  |  |  |  |  |  |  | 1 | 1 |  |  | 2 |
| **E** | 2 | 1 | 12 | 4 | 3 | 9 | 8 | 24 | 14 | 11 | 9 |  | 97 |
| **F** |  |  | 1 | 1 |  |  | 2 |  |  |  |  |  | 4 |
| **G** |  |  |  | 1 |  |  |  |  |  | 2 |  |  | 3 |
| **H** |  |  | 1 |  |  |  |  |  |  |  |  |  | 1 |
| **K** |  |  |  |  |  |  |  |  |  |  | 1 |  | 1 |
| **A+E** |  |  |  |  |  |  |  | 1 |  |  |  |  | 1 |
| **A+E+F** |  |  |  |  |  |  |  | 1 |  |  |  |  | 1 |
| **B+D+H** |  |  |  |  |  |  |  |  |  |  | 1 |  | 1 |
| **B+E** |  |  |  |  |  |  |  | 1 |  | 1 |  |  | 2 |
| **D+F** |  |  |  |  |  |  |  |  |  |  |  | 1 | 1 |
| **E+F** |  | 1 |  |  | 2 |  |  | 1 |  |  |  |  | 4 |
| **E+F+H** |  |  |  |  |  |  | 1 |  |  |  |  |  | 1 |
| **E+G** |  |  |  | 1 |  | 1 |  | 1 |  | 1 |  |  | 4 |
| **E+H** |  | 1 |  | 1 |  |  |  | 1 | 1 |  | 1 |  | 5 |
| **E+H+K** |  |  | 1 | 2 |  |  |  |  |  | 1 | 1 |  | 4 |
| **E+I** |  |  |  |  |  | 1 |  |  |  |  |  |  | 1 |
| **E+K** |  |  |  |  |  |  | 1 | 1 |  |  |  | 1 | 3 |

**The prevalence of *C. trachomatis* in Tunisian patients**

*sample that does not show *ompA* gene amplification and typing.
